# The Impact of Perceived Self‐Efficacy on Healthcare Transition Outcomes: Perceptions From Parents and Young People

**DOI:** 10.1111/cch.70125

**Published:** 2025-06-25

**Authors:** Cassandra Kwok, Daniel Waller, Michael Kohn, Frances L. Doyle

**Affiliations:** ^1^ School of Psychology Western Sydney University Sydney NSW Australia; ^2^ Transforming Early Education and Child Health Research (TeEACH) Research Centre Western Sydney University Sydney NSW Australia; ^3^ Academic Department of Adolescent Medicine The University of Sydney Children's Hospital Westmead Clinical School Sydney NSW Australia; ^4^ CRASH Centre for Adolescent's Health, Westmead Hospital Sydney NSW Australia; ^5^ School of Medicine Western Sydney University Sydney NSW Australia; ^6^ School of Psychological Science, Faculty of Medicine, Health and Human Science Macquarie University Sydney NSW Australia

**Keywords:** adolescents, chronic health, healthcare transition, parents, self‐efficacy, transition readiness

## Abstract

**Background:**

Adolescent and young adults (AYAs) with a chronic health condition face multiple challenges as they transition from paediatric to adult healthcare. To facilitate engagement during healthcare transition, one supportive psychological skillset is health self‐efficacy. Outcomes that indicate engagement during healthcare transition involve transition readiness, lower distress, quality of life and general adherence. Although researchers have examined the impact of youth self‐efficacy on engagement during healthcare transition, studies are yet to examine the impact of parent‐perceived self‐efficacy during healthcare transition. The current study aimed to investigate how youth self‐efficacy and parent‐perceived self‐efficacy impacted indicators of engagement during healthcare transition.

**Method:**

Participants were 54 AYAs and 48 parents who were recruited from The Centre for Adolescent and Young Adult Health at Westmead Hospital. Participating AYAs ranged in age from 12 to 25 years old (*M* = 17.74, *SD =* 2.56, *Mdn* = 17.08). Adolescents completed scales examining health self‐efficacy, distress, health‐related quality of life and general adherence to treatment. Parents completed scales examining AYAs' health self‐efficacy and transition readiness from paediatric to adult healthcare.

**Results:**

Uniquely, findings have demonstrated that parent‐perceived self‐efficacy holds most value in predicting transition readiness. Conversely, youth self‐efficacy holds most value in predicting general adherence.

**Conclusion:**

Both perspectives hold great importance for different outcomes. To promote successful healthcare transition and general adherence, self‐efficacy interventions that involve AYAs and parents would be beneficial.

**Summary**

- AYAs with chronic health conditions transitioning from paediatric to adult healthcare face many challenges.
- Self‐efficacy is one psychological skillset that may support AYAs during healthcare transition.
- Perspectives of self‐efficacy from both AYAs and parents are to be considered when examining indicators for engagement during healthcare transition.
- Findings indicated that parent‐perceived self‐efficacy holds most value in predicting transition readiness, while youth self‐efficacy holds most value in predicting general adherence.
- To promote successful healthcare transition and general adherence, self‐efficacy interventions that involve both AYAs and parents would be beneficial.

## 1 Introduction

Adolescent and young adults (AYAs) with chronic health conditions face multiple challenges as they transition from paediatric to adult healthcare. Unfortunately, many AYAs receive little to no preparation for the transition to adult healthcare. This can result in non‐compliance with treatment regimens and some AYAs dropping out of healthcare services (Wojciechowski et al. 2002; McDonagh and Viner 2006). One psychological skillset that facilitates engagement during healthcare transition is self‐efficacy beliefs, the perceived ability to perform behaviours in various situations (Bandura 1997; Colver et al. 2020). Health self‐efficacy is imperative for personal agency and provides people with the motivation to influence control over their lives (Bandura 1991; Anikputa et al. 2025). Health self‐efficacy is crucial during healthcare transition as it may assist AYAs in developing the knowledge and ability to manage their own chronic health condition (Rosen 1995; Varty et al. 2022). However, further research is required on how self‐efficacy relates to the emotional, psychological, and behavioural outcomes that indicate engagement during the transition to adult healthcare. Outcomes that indicate engagement in healthcare transition involve transition readiness, lower distress, quality of life and general adherence. To investigate self‐efficacy's impact on healthcare transition, self‐efficacy's relationship with each indicator will be explored in the following study.

### 1.1 Youth Self‐Efficacy

AYAs require self‐efficacy when faced with greater decision‐making responsibilities, increased accountability for their actions, relationship development, identity exploration, and changes in physical, social, and psychological functioning (Davis and Vander Stoep 1997). Those with chronic health conditions who are transitioning to adult healthcare face the added demands of becoming independent and responsible for the management of their health (Wojciechowski et al. 2002). Within the literature on healthcare transition, findings have indicated the beneficial impact of self‐efficacy on indicators of engagement during healthcare transition, such as transition readiness (Varty and Popejoy 2020; Torun et al. 2021), reduced distress (Clay and Telfair 2007), quality of life (Cramm et al. 2013; van Staa et al. 2011), and general adherence (Ravens et al. 2020). However, to date, there have been limited findings that demonstrate the impact of self‐efficacy on indicators of engagement during healthcare transition in a sample of AYAs undergoing transition from paediatric to adult healthcare.

Parents perspectives of their AYAs' self‐efficacy must also be considered because during healthcare transition, AYAs diagnosed with a chronic health condition have the tendency to rely heavily on their parents for healthcare management (van Staa et al. 2011). Meanwhile, parents worry most about whether AYAs have the self‐efficacy to independently manage their health condition (Boyle et al. 2001). There is preliminary evidence that suggests that parents may have differing perspectives of their AYAs' self‐efficacy in managing their health condition. However, there remains a lack of consensus on whether parents have difficulty ceding control of healthcare management to their AYAs, or have the tendency to either overestimate or underestimate their AYAs' self‐efficacy in managing their chronic health ^[13,1]^. Longitudinal findings have shown that increased self‐efficacy and appropriate parental involvement were strongly correlated with improved transition outcomes (Colver et al. 2018). One parental factor may be how confident parents are that their AYAs can manage their own health (i.e., parent‐perceived self‐efficacy). Parent‐perceived self‐efficacy is theorised to be important as (1) it may shape the behaviours that parents may enact, (2) parental behaviours are part of the environment for the AYAs and (3) environmental factors for the AYAs may also shape their own self‐efficacy. Parental perspectives of their AYAs' readiness to transition, wellness and perceived competence to look after themselves may also significantly impact on AYAs' progression to healthcare autonomy (Heath et al. 2017). During early stages of healthcare transition, AYAs have greater reliance on their parents compared to healthcare professionals. However, over time, they may reduce their parents' involvement in their care after successful healthcare transition and seek more support from healthcare professionals (Kennedy et al. 2007). This suggests that parents' perspectives may hold varying importance at different stages of the transition and with different healthcare transition outcomes. However, few studies have examined this. Hence, it is crucial to also consider how parent‐perceived self‐efficacy may influence outcomes (e.g., transition readiness, distress, quality of life and general adherence) during the critical period of healthcare transition.

Past findings suggest the importance of considering both AYAs' and parents' perspectives during healthcare transition (Heath et al. 2017). However, researchers still do not know how the perspectives of self‐efficacy between parents and AYAs differ in predicting healthcare outcomes. The present study focuses on bridging this gap by examining how youth and parent‐perceived self‐efficacy are associated with, and predictive of, indicators of engagement during healthcare transition such astransition readiness, reduced distress, quality of life and general adherence. It is of note that transition readiness was not reported by AYAs to reduce questionnaire burden. It was hypothesised that parent‐perceived self‐efficacy and AYA self‐efficacy would independently predict transition readiness, distress, quality of life and general adherence. Based on social cognitive theory (Bandura 1991), it was also hypothesised that parent‐perceived self‐efficacy and AYA self‐efficacy would interact to influence successful transition outcomes.

### 1.2 Centre for Adolescent and Young Adult Health (CAYAH)

CAYAH at Westmead Hospital is an outpatient facility that provides integrated care for AYAs transitioning from paediatric services at the Children's Hospital at Westmead to adult services at Westmead Hospital, Australia. CAYAH features a model of transition care for AYAs living with long‐term complex chronic health conditions (physical/mental health). This facility provides services that integrates medical, allied health and mental health professionals to address both medical and non‐medical difficulties faced by AYAs. All patients of CAYAH were eligible to participate; therefore, the age range of the participants was between 12 and 25. Patients enter CAYAH at varying ages between 12 and 25 based on their paediatric health team's determination with the intention of completing transition to adult services in 2 years.

## 2 Methods

### 2.1 Study Aim

The primary aim of the study is to examine whether youth and parent‐perceived self‐efficacy are associated with and predict transition readiness, distress, quality of life and general adherence.

### 2.2 Ethics

This study has received ethics committee approval from the Sydney Children's Hospital Network (2021/ETH11125) (Waller et al. 2024).

### 2.3 Measures

#### 2.3.1 Health Self‐Efficacy

##### 2.3.1.1 Youth Self‐Efficacy

The On Your Own Feet Self Efficacy Scale (OYOF‐SES) (Heath et al. 2017) is a self‐report questionnaire that measures disease‐related health self‐efficacy and was developed for AYAs transitioning from paediatric to adult healthcare (van Staa 2012). Participants rated 17 items on a 4‐point Likert scale ranging from 1 (*No, certainly not*) to 4 (*Yes, certainly*). Scores are summed together, and higher total scores indicate higher youth self‐efficacy to manage their chronic health condition. The 17‐item measure was chosen as it also demonstrates good validity and internal consistency amongst the subscales (Walter et al. 2018). Cronbach's alpha for this study was 0.89.

##### 2.3.1.2 Parent‐Perceived Self‐Efficacy

The On Your Own Feet Self‐Efficacy Scale–Parent (OYOF‐SES‐P) is a 17‐item questionnaire that was adapted from the OYOF‐SES. Parents rated items on a 4‐point Likert scale ranging from 1 (*no, certainly not*) to 4 (*yes, certainly*). Higher scores indicate greater youth self‐efficacy to manage their chronic health, as rated from the parents' perspective. Cronbach's alpha for this study was 0.96.

#### 2.3.2 Transition Readiness

The Readiness to Transition Questionnaire‐Parent (RTQ‐P) (Gilleland et al. 2012) assesses parents' perceptions of their AYAs' overall responsibility over healthcare behaviours and transition readiness. Similar to past research that measured overall transition readiness (Gilleland et al. 2012; Haarbauer‐Krupa et al. 2019), the two‐item Overall Transition Readiness subscale was used. Parents rated these items on a 4‐point Likert scale ranging from 1 (*Not responsible at all*) to 4 (*Almost always responsible*).

#### 2.3.3 Distress

The Kessler Psychological Distress Scale (K6) is a six‐item self‐report measure that is a global measure of distress (Kessler et al. 2003). Items were rated by the AYAs about symptoms experienced in the past 4 weeks on a 5‐point Likert scale, ranging from 1 (*None of the time*) to 5 (*All of the time*). The K6 has been found to have excellent psychometric properties (Furukawa et al. 2003). Scores range from 6 to 30. Scores between 6 and 18 reflect no probable serious mental illness, while scores between 19 and 30 reflect probable serious mental illness. Cronbach's alpha was 0.89.

#### 2.3.4 Quality of Life

The Patient‐Reported Outcomes Measurement Information System Quality of Life Measure (PROMIS‐10) is a 10‐item questionnaire that measures health‐related quality of life for the general population and patients with any disease condition, and represents the core health domains of physical health, pain, fatigue, mental health, social health and overall health (Cella et al. 2007). This questionnaire also demonstrates good construct validity with the RAND‐36, a health‐related quality of life measure (*r* = 0.81) (Hays et al. 2009). AYAs responded to items on a 5‐point Likert scale ranging from 1 (*Poor*) to 5 (*Excellent*). Scores are summed together, with higher scores indicating greater quality of life. Cronbach's alpha was 0.89.

#### 2.3.5 General Adherence

The Medical Outcomes Study (MOS) General Adherence Items is a five‐item self‐report questionnaire that measures general adherence to medical advice (Kravitz et al. 1993). AYAs rated how often they have adhered to medical advice over the past 4 weeks on a 6‐point Likert scale, ranging from 1 (*None of the time*) to 6 (*All of the time*). After reversing the scoring of items 1 and 3, scores are averaged to provide an overall rating of adherence. Cronbach's alpha was 0.85.

### 2.4 Procedure

AYAs and their parents accessing CAYAH were invited to participate either: (a) via email or (b) in‐person in the CAYAH waiting room (Waller et al. 2024). Data was collected at either the patient's initial or second appointment. Consent was collected from all participating AYAs and was required from a parent if the AYAs was under the age of 18. Informed consent was received from each participant and participating parent. Different consent forms were provided based on the AYAs' capacity to comprehend study information and provide informed consent. Next, participants completed all self‐report questionnaires on the online questionnaire platform REDCAP. Testing sessions took approximately 30 min. As compensation, participants were entered into a draw for gift card prizes (3 draws to the value of $250).

### 2.5 Statistical Analyses

Pearson correlations were used to examine the relationship between youth self‐efficacy, parent‐perceived self‐efficacy, transition readiness, distress, quality of life, general adherence and the control variables. Four hierarchical multiple regression models were also run on the outcome variables of transition readiness, distress, quality of life and general adherence. For each model, in step 1, the control variables of the AYAs' age and gender at birth were entered. In step 2, youth self‐efficacy was entered. In step 3, parent‐perceived self‐efficacy was entered. In step 4, the interaction between youth and parent‐perceived self‐efficacy were entered.

## 3 Results

Participants were 54 AYAs and 48 parents who were recruited from the Centre for Adolescent and Young Adult Health (CAYAH) at Westmead Hospital. There were 58 AYA‐parent dyads with different levels of missing data across the variables, but only 54 AYAs who have completed measures. CAYAH was selected because the clinic's population consists of parents and AYAs who are transitioning from paediatric to adult healthcare. See Table 1 for participant demographics. See Table 2 for descriptive statistics.

**TABLE 1:** Participant characteristics (*N* = 54).

	*n*	(%)
Adolescent or young adult age		
12–15 years	5	8.6
15–18 years	31	53.4
18–21 years	11	19.0
21–25 years	8	13.8
Missing	3	5.2
Parent age		
25–35 years	2	3.4
35–45 years	6	10.3
45–55 years	25	43.1
55–65 years	7	12.2
Missing	18	31.0
Gender at birth		
Female	30	51.7
Male	24	41.4
Prefer not to say	1	1.7
Missing	3	5.2
Current gender identity		
Female	29	50.0
Male	19	32.8
Transgender	3	5.2
Not listed, please specify		
Non‐binary	2	3.5
Questioning	1	1.7
Prefer not to say	1	1.7
Missing	3	5.2
Primary health condition		
Neurodevelopmental disorder	25	43.1
Eating disorder	17	29.3
Mental health disorder	4	6.9
Other	6	10.3
Missing	6	10.3
Highest level of education completed		
Some high school	26	44.8
High school	21	36.2
TAFE certificate	4	6.9
Some university	3	5.2
University degree	1	1.7
Missing	3	5.2
Currently working		
No	32	55.2
Yes, full‐time	5	8.6
Yes, part‐time	15	25.9
Prefer not to say	3	5.2
Missing	3	5.2
Speaks a language other than English		
No	42	72.4
Yes		
Turkish	3	5.2
French	2	3.4
Urdu	2	3.4
Persian	1	1.8
Hindi	3	5.2
Prefer not to say	2	3.4
Missing	3	5.2
Aboriginal or Torres Strait Islander		
No	48	82.8
Yes	6	10.3
Missing	4	6.9
Residential distance from the hospital		
Under 5 km	2	3.4
5 to 15 km	17	29.3
15 to 25 km	14	24.1
25 to 35 km	9	15.5
35 to 50 km	7	12.1
Over 50 km	3	5.2
Not sure	3	5.2
Missing	3	5.2

**TABLE 2:** Means, standard deviations and Pearson correlations.

Variable	*M*	*SD*	1	2	3	4	5	6	7	8
1. Youth self‐efficacy	50.9	8.7	—	—	—	—	—	—	—	—
2. Parent‐perceived self‐efficacy	45.5	11.8	0.21	—	—	—	—	—	—	—
3. Transition readiness	4.1	2.0	0.11	0.52***	—	—	—	—	—	—
4. Distress	10.2	5.5	−0.19	0.12	0.09	—	—	—	—	—
5. Quality of life	32.1	7.5	0.32*	0.09	−0.06	−0.80***	—	—	—	—
6. General adherence	4.1	1.0	0.53***	0.32*	0.12	−0.39**	0.39**	—	—	—
7. Patient age	17.6	2.6	0.47**	0.27	0.28	−0.00	0.16	0.31*	—	—
8. Gender at birth	—	—	0.03	0.01	0.01	0.40**	−0.34**	−0.18	0.06	—

*Note: M* and *SD* are used to represent mean and standard deviation, respectively. As all questionnaires used integers when measuring variables, only 1 decimal point was reported for *M* and *SD*.

*p < 0.05, **p < 0.01, ***p < 0.001

### 3.1 Correlations

As shown in Table 2, youth self‐efficacy was significantly, moderately and positively associated with quality of life and general adherence. However, youth self‐efficacy was not significantly correlated with transition readiness, nor with distress.

Parent‐perceived self‐efficacy was significantly, moderately and positively associated with transition readiness and general adherence. However, parent‐perceived self‐efficacy was not significantly correlated with quality of life, nor with distress.

### 3.2 Youth Self‐Efficacy and Parent‐Perceived Self‐Efficacy on Healthcare Outcomes

As shown in Table 3, greater transition readiness was predicted by parent‐perceived self‐efficacy, which explained 30% variance (*R* ^2^ = 0.30, adjusted *R* ^2^ = 0.21, *F*(5, 38) = 3.27, *p* = 0.015). For each one‐point change in parent‐perceived self‐efficacy, transition readiness increased by 0.08. Youth self‐efficacy and the interaction between youth and parent‐perceived self‐efficacy were not significant predictors of transition readiness.

**TABLE 3:** Model summary of hierarchical multiple regression for outcome variables of transition readiness, distress, quality of life and general adherence.

Outcome variable: transition readiness (*N =* 44)					
Step	*B (SE B)*	*β*	*t*	*R* ^2^ (adjusted *R* ^2^)	∆*R* ^2^
**Step 1**				0.08 (0.04)	0.08
Age	0.22 (0.12)	0.29	1.90		
Gender	−0.03 (0.56)	−0.00	−0.05		
**Step 2**				0.08 (0.01)	0.00
Age	0.23 (0.13)	0.30	1.73		
Gender	−0.03 (0.56)	−0.00	−0.05		
Youth self‐efficacy	−0.01 (0.04)	−0.03	−0.15		
**Step 3**				0.30 (0.23)**	0.22**
Age	0.15 (0.12)	0.19	1.24		
Gender	−0.02 (0.50)	−0.00	−0.04		
Youth self‐efficacy	−0.02 (0.04)	−0.08	−0.50		
Parent‐perceived self‐efficacy	0.08 (0.02)	0.49**	3.47		
**Step 4**				0.30 (0.21)*	0.00
Age	0.14 (0.12)	0.18	1.16		
Gender	0.01 (0.51)	0.00	0.02		
Youth self‐efficacy	−0.02 (0.04)	−0.08	−0.51		
Parent‐perceived self‐efficacy	0.08 (0.03)	0.50**	3.39		
Youth self‐efficacy × parent self‐efficacy	0.00 (0.00)	0.06	0.39		

Outcome variable: distress (*N = 44*)					
Step	*B* (SE *B*)	*β*	*t*	*R* ^2^ (adjusted *R* ^2^)	∆*R* ^2^
**Step 1**				0.16 (0.12)*	0.16*
Age	−0.06 (0.31)	−0.03	−0.21		
Gender	4.12 (1.47)	0.40**	2.81		
**Step 2**				0.21 (0.15)*	0.05
Age	0.19 (0.34)	0.09	0.54		
Gender	4.13 (1.44)	0.40**	2.86		
Youth self‐efficacy	−0.15 (0.10)	−0.25	−1.54		
**Step 3**				0.23 (0.15)*	0.02
Age	0.12 (0.35)	0.06	0.34		
Gender	4.14 (1.44)	0.40**	2.87		
Youth self‐efficacy	−0.16 (0.10)	−0.26	−1.63		
Parent‐perceived self‐efficacy	0.07 (0.07)	0.15	1.02		
**Step 4**				0.23 (0.13)	0.00
Age	0.13 (0.36)	0.06	0.36		
Gender	4.08 (1.48)	0.40**	2.76		
Youth self‐efficacy	−0.16 (0.10)	−0.26	−1.60		
Parent‐perceived self‐efficacy	0.06 (0.07)	0.14	0.89		
Youth self‐efficacy × parent self‐efficacy	−0.00 (0.01)	−0.04	−0.24		

Outcome variable: quality of life (*N =* 44)					
Step	*B* (SE B)	*β*	*t*	*R* ^2^ (adjusted *R* ^2^)	∆*R* ^2^
**Step 1**				0.20 (0.16)*	0.20*
Age	0.39 (0.41)	0.14	0.96		
Gender	−6.00 (1.96)	−0.43**	−3.06		
**Step 2**				0.27 (0.22)**	0.08*
Age	−0.04 (0.45)	−0.02	−0.10		
Gender	−6.01 (1.88)	−0.43**	−3.19		
Youth self‐efficacy	0.27 (0.13)	0.32*	2.07		
**Step 3**				0.28 (0.20)*	0.00
Age	−0.08 (0.46)	−0.03	−0.18		
Gender	−6.00 (1.90)	−0.43**	−3.16		
Youth self‐efficacy	0.27 (0.13)	0.31	1.99		
Parent‐perceived self‐efficacy	0.04 (0.09)	0.06	0.45		
**Step 4**				0.28 (0.18)*	0.00
Age	−0.01 (0.47)	−0.04	−0.22		
Gender	−5.92 (1.95)	−0.42**	−3.04		
Youth self‐efficacy	0.26 (0.14)	0.31	1.96		
Parent‐perceived self‐efficacy	0.05 (0.10)	0.08	0.51		
Youth self‐efficacy × parent self‐efficacy	0.00 (0.01)	0.04	0.29		

Outcome variable: general adherence (*N* = 44)					
Step	*B* (SE *B*)	*β*	*t*	*R* ^2^ (adjusted *R* ^2^)	∆*R* ^2^
**Step 1**				0.14 (0.10)*	0.14*
Age	0.13 (0.06)	0.33*	2.23		
Gender	−0.39 (0.28)	−0.20	−1.37		
**Step 2**				0.33 (0.28)**	0.19**
Age	0.04 (0.06)	0.09	0.62		
Gender	−0.39 (0.25)	−0.20	−1.55		
Youth self‐efficacy	0.06 (0.02)	0.49**	3.35		
**Step 3**				0.37 (0.30)**	0.04
Age	0.02 (0.06)	0.05	0.30		
Gender	−0.39 (0.25)	−0.20	−1.57		
Youth self‐efficacy	0.06 (0.02)	0.47**	3.25		
Parent‐perceived self‐efficacy	0.02 (0.01)	0.22	1.62		
**Step 4**				0.37 (0.29)**	0.00
Age	0.02 (0.06)	0.06	0.36		
Gender	−0.40 (0.25)	−0.21	−1.61		
Youth self‐efficacy	0.06 (0.02)	0.47**	3.23		
Parent‐perceived self‐efficacy	0.02 (0.01)	0.20	1.40		
Youth self‐efficacy × parent self‐efficacy	0.00 (0.00)	−0.06	−0.46		

*Note:* Gender refers to gender at birth.

*p < 0.05, **p < 0.01, ***p < 0.001

The model predicting distress was not significant. However, distress was independently predicted by gender. On average, female AYAs reported more distress than males AYAs by 4.08. Youth self‐efficacy, parent‐perceived self‐efficacy, and their interaction were not significant predictors of distress.

Greater quality of life was predicted by gender, which explained 28% variance (*R* ^2^ = 0.28, adjusted *R* ^2^ = 0.18, *F*(5, 38) = 2.93, *p* = 0.025). On average, female AYAs reported lower quality of life than male AYAs by 5.92. Youth self‐efficacy, parent‐perceived self‐efficacy, and their interaction were not significant predictors of quality of life.

Greater general adherence was predicted by youth self‐efficacy, which explained 37% variance (*R* ^2^ = 0.37, adjusted *R* ^2^ = 0.29, *F*(5, 38) = 4.51, *p* = 0.003). For each one‐point change in youth self‐efficacy, general adherence increased by 0.06. Parent‐perceived self‐efficacy and the interaction between youth and parent‐perceived self‐efficacy were not significant predictors of general adherence.

## 4 Discussion

The aim of the present study was to investigate how youth self‐efficacy and parent‐perceived self‐efficacy are associated with indicators of engagement during healthcare transition. In accordance with previous research (Cramm et al. 2013; Ravens et al. 2020), higher youth self‐efficacy was associated with higher quality of life, greater general adherence and older age. However, youth self‐efficacy was not significantly associated with parent‐perceived self‐efficacy, transition readiness, distress or gender at birth. It is possible that youth self‐efficacy was not related to parents' perceptions of their AYAs' self‐efficacy because parents may not hold a holistic understanding of their AYAs' self‐efficacy. An alternative explanation may be that youth may interpret questions about self‐efficacy differently to their parents. Past research has indicated that parents tend to overestimate or underestimate AYAs' self‐efficacy (Cramm et al. 2013; Sonneveld et al. 2013). Additionally, it is possible that youth self‐efficacy was not related to transition readiness as this outcome was rated by parents rather than AYAs. Previous studies reporting significant findings have used self‐report to measure transition readiness (Varty and Popejoy 2020). It is possible that there could be shared method variance inflating associations when both measures are AYAs self‐reported. Moreover, it is possible that the relationship between self‐efficacy and distress differs in the present study as patients in the present sample predominantly have primary mental health conditions. Previous studies often focused on samples with primary physical health conditions (Varty and Popejoy 2020; Torun et al. 2021).

In accordance with previous research (Speller‐Brown et al. 2015; Croom et al. 2011), higher parent‐perceived self‐efficacy was associated with greater transition readiness and general adherence. However, parent‐perceived self‐efficacy was not significantly associated with distress, quality of life, age or gender at birth. It is possible that parent‐perceived self‐efficacy was not related to distress because the study's sample involved a range of chronic conditions. Previous research has focused on samples with only one chronic condition. However, the current sample has differed from previous research as it includes AYAs with physical and/or mental health conditions such as eating disorders and neurodevelopmental disorders. It is possible that these results may generalise more broadly to adolescents with chronic conditions rather than on a specific cohort (Clay and Telfair 2007) Additionally, it is possible that parent‐perceived self‐efficacy was not related to quality of life because quality of life was rated by the AYAs themselves. In previous research (Uzark et al. 2019; Uzark et al. 2020) parents of AYAs below the age of 18 would complete questionnaires on behalf of their adolescent.

This study also aimed to examine whether youth and parent‐perceived self‐efficacy significantly predicted successful healthcare transition outcomes. It was hypothesised that parent‐perceived self‐efficacy and youth self‐efficacy would independently predict transition readiness, distress, quality of life and general adherence. Results showed that greater general adherence was independently predicted by higher youth self‐efficacy. Further, greater transition readiness was independently predicted by higher parent‐perceived self‐efficacy. Neither youth or parent‐perceived self‐efficacy independently predicted distress or quality of life. This pattern of results demonstrate that different perspectives hold varying importance for different healthcare transition outcomes. Different perspectives are crucial to ensure successful healthcare transition (Loecher et al. 2023). During healthcare transition, all stakeholders play an important role and hold different perspectives (Varty and Popejoy 2020). When parents are involved in healthcare transition through sharing their perspectives, they are also able to closely observe their AYAs' ability to manage their health condition (Schwartz et al. 2013). This may be associated with greater parental confidence that their AYAs is ready for healthcare transition (Ellison et al. 2022). Additionally, consideration of both AYAs' and parents' perspectives fosters strategies that support health self‐management, promotes treatment adherence, and facilitates goal setting towards successful healthcare transition (Jiang et al. 2021).

Additionally, based on social cognitive theory (Bandura 1991), it was hypothesised that parent‐perceived self‐efficacy and youth self‐efficacy would interact to influence successful transition outcomes. However, there was no evidence for an interaction in any of the models. Thus, empirical data does not support this theorised relationship.

Gender at birth and age were entered into all models as a control variable as previous research has repeatedly shown these relationships influence the outcome measures (Rucklidge and Tannock 2001). Age was not found to be an independent predictor in any of the models. Similarly, gender was not significantly predictive of transition readiness nor general adherence. However, gender at birth was shown to be a significant predictor of distress and quality of life. On average, females reported more distress and lower quality of life than males. This aligns with past findings amongst samples of neurodevelopmental disorders, eating disorders and chronic conditions (Rucklidge and Tannock 2001; Bentley et al. 2015; Petersen et al. 2006).

Based on the current study's findings, obtaining different perspectives of self‐efficacy holds value in predicting different facilitators for successful healthcare transition. During healthcare transition, both AYAs' and parents' perspectives of self‐efficacy are needed (Singh et al. 2018). Clinicians and practitioners may benefit in considering parent‐perceived self‐efficacy when facilitating transition readiness. Whereas, clinicians and practitioners may benefit in considering youth self‐efficacy when supporting general adherence to treatment. Furthermore, self‐efficacy may be targeted for intervention to foster successful healthcare transition (as measured through general adherence and transition readiness). Self‐efficacy is a malleable skill that is multidetermined by many sources (Bandura 1991). Based on social cognitive theory (Bandura 1991), to construct self‐efficacy, people synthesise information from mastery experiences (e.g., independent management of health), vicarious experience (e.g., observing others manage their health), verbal persuasion (e.g., parents verbally supporting better AYA health behaviours), and physiological states (e.g., positive physiological sensations). Hence, interventions for AYAs and parents that foster youth self‐efficacy may support healthcare transition.

Whilst this study adds to the literature, there are also limitations. First, the study was cross‐sectional. Thus, temporal ordering and causality could not be determined. Second, shared method variance may be influencing results as transition readiness and parent‐perceived self‐efficacy were both parent‐reported. Whilst researchers have highlighted the importance of obtaining parents perspectives during healthcare transition (Heath et al. 2017), future research may wish to employ clinician ratings to determine healthcare transition outcomes. Third, this study employed several self‐report measures. Whilst self‐efficacy must be self‐reported (Burrell et al. 2018), other outcome variables could be obtained via observation or other behavioural‐based data that may be extracted from electronic medical records. Fourth, the current study could have greater representation of a range of primary physical chronic health conditions. Fifth, transition readiness was not reported by AYAs to reduce questionnaire burden. Future research may wish to investigate valid and reliable measures for transition readiness reported by AYAs. Sixth, as self‐report measures were completed during early appointments at CAYAH, the age of participants when the measures were completed may significantly affect perceived self‐efficacy and/or transition readiness.

This study also had many strengths. A key strength is that the study was conducted at CAYAH, a novel healthcare service for AYAs undergoing healthcare transition. Current findings also extend the literature by examining the role of self‐efficacy across various healthcare outcomes in patients with a variety of chronic conditions. Moreover, this is the first study to consider both AYA and parental perspectives of self‐efficacy in relation to indicators of engagement during healthcare transition. Finally, majority of the study's cohort presented with chronic mental health difficulties, as opposed to chronic physical illness. The findings from the present study further extend the literature by considering mental health transition amongst AYAs.

In conclusion, this study aimed to investigate how youth self‐efficacy and parent‐perceived self‐efficacy impacted indicators of engagement during healthcare transition. Uniquely, findings have demonstrated that parent‐perceived self‐efficacy holds most value in predicting transition readiness. Conversely, youth self‐efficacy holds most value in predicting general adherence. Thus, both perspectives hold great importance for different outcomes. To promote successful healthcare transition and general adherence, self‐efficacy interventions that involve AYAs and parents could be beneficial.

## Author Contributions

**Cassandra Kwok:** investigation, methodology, formal analysis, project administration, writing – review and editing, writing – original draft, data curation, software, validation, visualization. **Daniel Waller:** supervision, conceptualization, funding acquisition, project administration, methodology, writing – review and editing, validation, software, visualization. **Michael Kohn:** supervision, writing – review and editing, validation, visualization, resources. **Frances L. Doyle:** writing – review and editing, supervision, validation, visualization.

## Ethics Statement

This study has received ethics committee approval from the Sydney Children's Hospital Network (2021/ETH11125) with site‐specific approvals from the WSLHD (2021/STE03184) and the SCHN (2023/STE00977).

## Conflict of Interests

The authors declare no conflicts of interest.

## Data Availability

The data that support the study's findings are available from the authors, upon reasonable request.
